# The Present and Future of Scientific Publications

**Published:** 1893-06

**Authors:** 


### Editorial.

### THE PRESENT AND FUTURE OF SCIENTIFIC PUBLICATIONS.

We are dwelling in an age of rapid thought. The steps that lead to the pinnacle of endeavor are not taken with slow and rhythmical precision, but with leaps and bounds, so quickly made that to the ordinary mind they are confusing in their rapidity and far-reaching power.

From the date when Gutenberg invented movable types, the thought has possessed the mind of the world that the only proper and permanent mode of communicating ideas was through the medium of books, and these have multiplied into libraries, and libraries have extended into every hamlet of the civilized world. To the bibliophile this is a very satisfactory condition. The larger the number of books the more conclusive the evidence of an advance in civilization.

In a purely literary sense there can be no controversy with this feeling. The work of the ages lives in the volumes of the libraries, and the world would be poor indeed were it possible by a cataclysm to blot this accumulated thought and experience from the face of the earth.

While recognizing this universally-accepted truth, it is with no iconoclastic spirit that the question is prominently asked, Have books, and especially scientific books, ceased to have special value as a means for higher training? The interrogatory is too broad a one to be answered in an editorial article, nor perhaps can it be replied to satisfactorily anywhere. The influences which dominate mental training are of such a subtle character that the search becomes illusive and conclusions may be warped by misdirected thought. There are, however, certain avenues which may be explored with some degree of assurance of not being led into a labyrinth of metaphysical disquisitions and uncertain conclusions.

Books are the depositaries of the world's wisdom; they can never illustrate its active progress, for as soon as any advance enters into permanent form, embalmed, as it were, in boards and leather, it becomes history, and history is simply the record of dead activities.

When we examine the shelves of our scientific libraries it is with a feeling akin to that with which we observe a collection of antiques. They hold the memorials of a dead past, and are practically of no real use. Each of these tomes has served its purpose. It is a link in the educational chain, but at present is only one piece in the monument erected to commemorate a world's advancement from barbaric thought.

It was said of the late Dr. Atkinson that he rejoiced that he had never been so foolish as to write a book, and it was Agassiz who persistently refused to permit his students to study from books. Both of these prominent laborers in widely diverse fields of investigation were right and wrong. Right in the motive, but wrong in the inference seemingly sought to be inculcated, that books were of no value. Their value in a scientific sense, as it appears to the writer, cannot be overestimated, but the mind that places reliance upon them will fail to grow beyond the parasitical stage, depending for its mental aliment upon the work of others. It must, thus trained, necessarily be in a condition of permanent childhood, advancing only as it is led.

Books are of two classes, original and compiled. The former live by divine right, and will continue to enlighten the world long after their effulgence has been dimmed by more brilliant discoveries. The latter die almost with the issuing. They can only exist as text-books, and as such must be renewed constantly by revision, and then they fail in the end to contain the latest original thought, and are to that extent useless. All are familiar with these two classes. The original production is so rare that its existence may be questioned, yet the truly investigating mind surrounds even old things with a new flavor, and we would not willingly let these die. Then add to these the discoveries of this same thinker, and the work is handed down the ages.

The impossibility of binding the advancing thought of this period in volumes is nowhere more apparent than in our own profession. It has been repeatedly attempted, but outside of special lines of work it has been a failure. We hand these books to our students with a mental reservation, wondering if we are not doing them a positive wrong in so doing.

All rapidly-growing scientific thought requires an equally rapid medium of transmission. Books are proverbially slow in development. Two or three years in process of growth from the possibly crudely-formed ideas in the author's brain to the hands of the reader is an age in the development of scientific work. This may be well illustrated in the advance made in the study of electricity. Twelve years ago electrical lighting was in its infancy, and the dynamo was to the laity an unknown instrument of power. To-day it is difficult to keep pace with the marvellous developments in this direction. No printing-press is equal to recording them in books, and so astounding are they in some of their phases, that the mind is simply stunned by the momentous possibilities of the future. When these rapid strides in one specialty are considered, how incomparably tame seems the ordinary text-book!

In recent years we have been afflicted with a deluge of small books, and this is doubtless the result of the before-mentioned fact that large books have practically proved failures. In a condensed and frequently-issued form it is presupposed that the student will be amply provided with more recent original investigations. If this idea has had anything to do with their production, it has not been an unmixed success. Condensed thought is rarely satisfactory, whether printed or verbal. There is a happy mean between prolixity and loquacity and sententious and concise expression that but few of us are equal to attaining, and, therefore, the small book is rarely a scientific success, if indeed it be not a misleading production, conducting the untrained mind into confused and impracticable ideas.

The future processes for embalming scientific thought in its most advanced form may be open to conjecture. It cannot be relegated to ephemeral publications. The journals, whether weekly, monthly, or quarterly, furnish an excellent substitute until something better may be devised; yet these can never fill a desired want in educational processes. When the small book is published, covering only one subject, compact and yet full, embracing everything new, and issued yearly, we will have approached, in the writer's opinion, very nearly to the ideal work. Until this be accomplished, we must be satisfied with the cruder educational facilities of the older and slower civilizations.

